# Minimally Invasive Aesthetic Treatments for the Corrective Patient Archetype: Transforming Lives

**DOI:** 10.1007/s00266-026-05747-w

**Published:** 2026-04-16

**Authors:** Isaac Shturman, Hamilton Garzón, Daniel Coimbra, Jorge Villafranca, Silvia Zimbres, Maria de los Angeles Pyke, Irina Van Der Graaff, Maria Luiza Dabronzo

**Affiliations:** 1 Plastica Shturman, Hospital Angeles Lomas, Vld de La Barranca S/N, 01000 Ciudad de México, DF, Estado de México México; 2 Clínica Antiedad, Bogotá, Colombia; 3 Instituto de Dermatologia Professor Rubem David Azulay, Santa Casa de Misericórdia/IDPRDA, Rio de Janeiro, Brazil; 4 Private Practice, Santiago, Chile; 5 Clinica Doux, São Paulo, Brazil; 6 Allergan Aesthetics, An AbbVie Company, Buenos Aires, Argentina; 7 Allergan Aesthetics, An AbbVie Company, Mexico City, Mexico; 8 Allergan Aesthetics, An AbbVie Company, São Paulo, Brazil

**Keywords:** Botulinum toxin, Hyaluronic acid, Aesthetics, Facial reconstruction, Facial correction

## Abstract

**Background:**

The definition of health encompasses mental, emotional, and social well-being, rather than simply the absence of disease. As a result, corrective aesthetic treatments are increasingly being viewed as an integral component of overall health rather than confined solely to the domain of aesthetics. In the realm of facial aesthetics, the corrective patient archetype is distinctly focused on rebalancing or proportioning facial features to improve confidence and self-perception. Minimally invasive treatments, such as botulinum toxin and hyaluronic acid (HA) gels, are known for their efficacy, tolerability, and ability to improve psychological and social outcomes. Additionally, hybrid injectables are gaining traction for their immediate filling and lifting effects that also provide biostimulating effects over time in the injected area.

**Methods:**

Here, we present a clinical review of the literature, accompanied by examples that illustrate the practical application of onabotulinumtoxinA, HA gels, and hybrid injectables (HA and calcium hydroxyapatite; Allergan Aesthetics, an AbbVie Company) for patients seeking corrections, including facial asymmetries (e.g., facial paralysis and hemifacial microsomia), trauma, and scarring, emphasizing the importance of tailored approaches to achieve optimal outcomes.

**Results and Conclusions:**

OnabotulinumtoxinA, HA gels, and hybrid injectables were effective, tolerable, and enhanced patients’ quality of life by addressing both aesthetic and functional concerns. Overall, further research is needed to refine these treatments and expand their applications in corrective aesthetic medicine.

**Level of Evidence IV:**

This journal requires that authors assign a level of evidence to each article. For a full description of these Evidence-Based Medicine ratings, please refer to the Table of Contents or the online Instructions to Authors www.springer.com/00266.

## Introduction

### Patient Archetypes

Four primary patient archetypes in aesthetic medicine have been proposed by Liew et al: beautification, positive aging, transformation, and correction [1]. Patients in the beautification, positive aging, and transformation archetypes are mainly focused on aesthetics, while patients in the correction archetype are driven by a desire to improve a particular feature that they believe has a negative impact on their life [1]. Corrective patients seek to rebalance facial features for improved confidence and self-perception. Features that may be considered bothersome by patients in the correction archetype vary, but are typically congenital or acquired through trauma, surgery, or medical illness [1]. This manuscript offers detailed insights on corrective treatments, including examples of common circumstances that may require corrective facial procedures, and includes illustrative clinical cases.

### Minimally Invasive Procedures for the Corrective Patient Archetype

Clinical experience with facial injectable products, including onabotulinumtoxinA (Allergan Aesthetics, an AbbVie Company, Irvine, CA) and hyaluronic acid (HA) gels, is extensive [2–6]. OnabotulinumtoxinA has become the standard of care in multiple indications [7–11], elevating patient care, fulfilling unmet needs, and changing treatment paradigms due to its established efficacy and tolerability [12–19]. HA gel products have been used successfully for aesthetic improvements of the nose, infraorbital hollows, dynamic radial cheek lines, hands, neck, and décolletage [20–24]. Together, onabotulinumtoxinA and HA gels are the 2 most commonly used nonsurgical treatments worldwide [25, 26]. Additionally, novel hybrid injectables can enhance patient satisfaction with treatment by instantly restoring volume and boosting collagen production, thereby enhancing skin structure and soft tissue quality over time [27–29]. The corrective patient archetype is likely to obtain psychological (including self-perception, self-confidence, and social perception) and social benefits from facial aesthetic treatments, along with improvements in self-perceived appearance and age [1, 30, 31], further highlighting the importance of appropriate treatment in these patients.

### Clinical Circumstances for the Corrective Patient Archetype

Facial injectables have been used to effectively correct a variety of concerns and areas of the face and neck, including restoration of facial symmetry and correction of facial trauma and scaring. A summary of relevant, available reports is discussed below.

#### Facial Asymmetry

##### Facial Paralysis

Botulinum toxin is a key tool for managing facial hyperkinetic lines and the effects of facial paralysis, addressing functional deficits, and enhancing patients’ quality of life [32, 33]. Similarly, myomodulation with HA gels has shown benefits in patients with facial paralysis and associated sequelae [34, 35], where the potential ability of HA gels to modulate muscle activity has been demonstrated to enhance muscle balance and function, resulting in improved aesthetic outcomes and improved quality of life for patients with facial paralysis [36]. Periocular injections with HA gels have been observed to enhance chin and perioral dynamics, with effects being maintained for well over 1 year in a case study examining long-standing facial paralysis and sequelae [34]. A multimodal treatment approach has also shown benefit for those with facial paralysis, as a case study using botulinum toxin, HA gel, and thread lifting found that, despite long disease duration and advanced age, a satisfactory treatment result was obtained that markedly improved the patient’s self-esteem [37]. In corrective patients, especially those with altered anatomy from prior surgery, tailoring the rheological profile (e.g., high G´ and high cohesivity for lift or medium G´ for muscle modulation) can achieve precise outcomes like restoring volume, improving muscle control, and enhancing skin mechanics in complex facial landscapes.

##### Hemifacial Microsomia

In cases of hemifacial microsomia, the severity of deformity on the affected side varies substantially among patients [38]. Consequently, the timing and approach to intervention must be tailored to each patient’s unique circumstances and needs and may involve skeletal, dental, and soft tissue components [38]. Early intervention in hemifacial microsomia is crucial for optimizing outcomes and minimizing deformities [38]. HA gels have been successfully used to manage patients with hemifacial microsomia, but a complex treatment approach may be necessary for these patients given the expressive asymmetry that can occur [1].

#### Facial Trauma and Scarring

##### Facial Trauma

Skin that is damaged by trauma or surgical intervention will inevitably generate scars, with facial scars impacting psychological well-being, especially as they widen over time [39]. HA gels can be a successful tool for patients who have a facial trauma by restoring volume and potentially stimulating collagen production [40]. Multiple studies have found HA gels to be effective in treating traumatic atrophic depressed facial scarring [41, 42]. To assess the suitability of scars for HA gel treatment, the “dimple sign” can be employed [42]. This method involves applying lateral inward pressure on the skin; if the pressure induces a depression (dimple) due to the scar’s attachment to the underlying subcutaneous tissue (positive dimple sign), HA gel alone may not be effective [42]. In such cases, alternative treatments like subcision, excision, or laser therapy may be needed [42].

##### Surgical Resection of Skin Cancers

After major reconstructive surgeries, aesthetic improvements with HA gels are particularly beneficial, as patients undergoing complex facial reconstructions often avoid secondary procedures to improve scarring [43]. A case study found that after postsurgical resection of a squamous cell carcinoma on the cheek requiring a skin graft, HA gel treatment resulted in progressive volumization of the area [43]. Additionally, the initially immobile skin graft showed progressive improvement in softness and mobility, and stable results were observed in volumization and softness of the treated area 9 months postinjection [43]. A separate case report found that postsurgical depressed scars in patients who underwent Mohs surgery for basal cell carcinoma were effectively mitigated with HA gel [44]. As not all HA gels are suitable for all types of corrections due to differences in biochemical properties, studies are needed to determine the appropriate choice for each clinical condition [43]. HA gels could become a convenient, long-lasting solution to improve postsurgical facial deformities and scars after skin cancer resections [43].

## Materials and Methods

### Scope

This manuscript presents a descriptive clinical review with illustrative examples examining the use of minimally invasive injectable treatments in patients within the corrective patient archetype. The clinical examples were selected to demonstrate representative corrective scenarios encountered in aesthetic practice, including facial asymmetry related to congenital conditions, paralysis, trauma, and scarring.

### Injectable Products

Minimally invasive treatments included onabotulinumtoxinA, HA gels (VYC-15L, VYC-17.5L, VYC-20L, and/or VYC-25L), and, where clinically appropriate, a hybrid injectable composed of HA and calcium hydroxyapatite (all products from Allergan Aesthetics, an AbbVie Company, Irvine, CA). Product selection was individualized based on anatomical considerations, degree of asymmetry, tissue quality, and treatment objectives. Healthcare professionals reviewed the approved indications for use of each product, recognizing that regulatory approvals may vary by country.

### Treatment Planning

Treatment planning was guided by a comprehensive facial assessment, including evaluation of static and dynamic asymmetry, muscle activity, soft tissue volume deficits, skeletal support, and any prior surgical, traumatic, or disease-related alterations in anatomy. Particular attention was paid to differences between hyperactive and hypofunctional muscle groups, tissue stiffness, and regional vascular considerations in corrective patients.

### Injection Techniques

Injection techniques were selected based on the anatomical region, treatment goal, and tissue characteristics. OnabotulinumtoxinA, when used, was administered intramuscularly to address hyperkinetic muscle activity contributing to facial imbalance or asymmetry. Dosing and injection sites were individualized, and asymmetric dosing was employed when necessary to achieve functional and aesthetic balance.

HA gels and hybrid injectables were placed using a combination of supraperiosteal, deep subcutaneous, suborbicular, and superficial subdermal techniques, depending on the indication. Structural support and volume restoration were achieved through deep-plane placement, while superficial placement was used selectively for contour refinement or myomodulation. Both needles and blunt-tip cannulas were used according to anatomical region, vessel proximity, and clinician judgment. Injection techniques included linear retrograde, bolus, fanning, columnar, and retro injection methods, as appropriate for the corrective objective.

### Treatment Delivery

Treatments were administered in single or multiple sessions depending on clinical complexity and patient needs. The sequence and combination of injectable products were individualized, and treatments were performed with standard aseptic technique. Injection volumes, planes, and session frequency were adjusted to minimize risk while optimizing corrective outcomes.

### Documentation and Safety Monitoring

Standardized clinical photographs were obtained before and after treatment to document outcomes. Patients were monitored for adverse events during and following procedures, and follow-up assessments were conducted according to routine clinical practice. Any adverse events were recorded.

### Ethical Considerations

All patients provided written informed consent for treatment and for the use of clinical images for educational and publication purposes. Treatments were performed in accordance with local regulations and standard clinical practice. This work did not involve experimental interventions, prospective enrollment, or systematic data collection.

## Results

The corrective patient archetype has been successfully clinically treated with HA gels, hybrid injectables, and/or onabotulinumtoxinA, resulting in improved patient satisfaction, facial symmetry, and facial contour and balance (Table 1). Clinical cases are discussed in detail below. Healthcare professionals must review the indications for use of each of the products used, as they may vary in different countries.

**Table 1:** Overview of presented treatments and outcomes

Clinical Example	Patient Details	Reason for Treatment	Treatments	Outcomes
*Facial asymmetry*				
1	44-year-old White male, Fitzpatrick skin type III	Facial paralysis (post–herpes zoster infection)	HA gels (VYC-15L, VYC-17.5L, VYC-20L, VYC-25L) and hybrid injectables (HA and calcium hydroxyapatite); onabotulinumtoxinA	Substantial improvement in facial symmetry (including dynamic movements); no adverse events
2	58-year-old Hispanic female, Fitzpatrick skin type III	Facial paralysis (congenital)	HA gels (VYC-17.5L, VYC-20L, VYC-25L); onabotulinumtoxinA	Improved facial symmetry; no adverse events
3	20-year-old White female, Fitzpatrick skin type III	Hemifacial microsomia type I	HA gels (VYC-15L, VYC-20L, VYC-25L)	Improved facial symmetry; no adverse events
*Facial trauma and scarring*				
5	44-year-old Hispanic female, Fitzpatrick skin type IV	Synovial sarcoma sequelae	HA gels (VYC-15L, VYC-17.5L, VYC-20L)	Improved facial symmetry and contour; no adverse events
6	44-year-old White female, Fitzpatrick skin type III	Facial trauma: bicycle accident	HA gels (VYC-15L, VYC-17.5L, VYC-20L, VYC-25L)	Improved facial symmetry (including dynamic movements), mild bruising resolved spontaneously

*HA* hyaluronic acid

### Facial Asymmetry

#### Correction of Facial Paralysis After Herpes Zoster Infection

A White man 44 years of age with Fitzpatrick skin type III presented with facial paralysis after a herpes zoster infection. He sought treatment options 1 year after the onset of facial paralysis. The clinical examination revealed incomplete movements of the left face affecting all 3 thirds of the face, synkinesis, hemifacial spasms, and sequelae of paralysis. The patient had no comorbidities and had previously undergone physiotherapy.

HA gels (VYC-15L, VYC-17.5L, VYC-20L, and VYC-25L; Allergan Aesthetics, an AbbVie Company) and a hybrid injectable (HA and calcium hydroxyapatite; Allergan Aesthetics, an AbbVie Company) were used to restore facial symmetry. On the affected side (left), 1.25 mL of the hybrid injectable was placed into the zygomatic and preauricular areas, 1.0 mL VYC-20L into the posterior temple, 0.5 mL VYC-20L into the anterior temple, 0.5 mL VYC-20L into the malar area, 0.2 mL VYC-20L into the pyriform fossa, and 0.7 mL VYC-15L into the tear trough. On the unaffected side (right), 1.25 mL of the hybrid injectable was injected into the zygomatic and preauricular areas, 0.7 mL VYC-20L into the malar area, 0.3 mL VYC-20L into the pyriform fossa, and 0.6 mL VYC-15L into the tear trough. The chin was treated with 1.0 mL of VYC-20L, 2.0 mL of VYC-25L, and 1.0 mL of VYC-17.5L. These HA gels were administered in a single treatment session.

Additionally, onabotulinumtoxinA was administered intramuscularly using a 31-G needle for the treatment of asymmetrical hyperkinetic facial lines. The anatomical areas treated included the glabella (20 U), orbicularis oculi (22 U), frontalis muscle on the right side (6 U), nasal area (3 U), levator labii superioris alaeque nasi (1 U), mentalis (8 U), and platysma (40 U). The total dose was 100 U, diluted in 2 mL of saline solution. The onabotulinumtoxinA treatments were repeated approximately once every 5 months over a 2-year period.

**Fig. 1:**
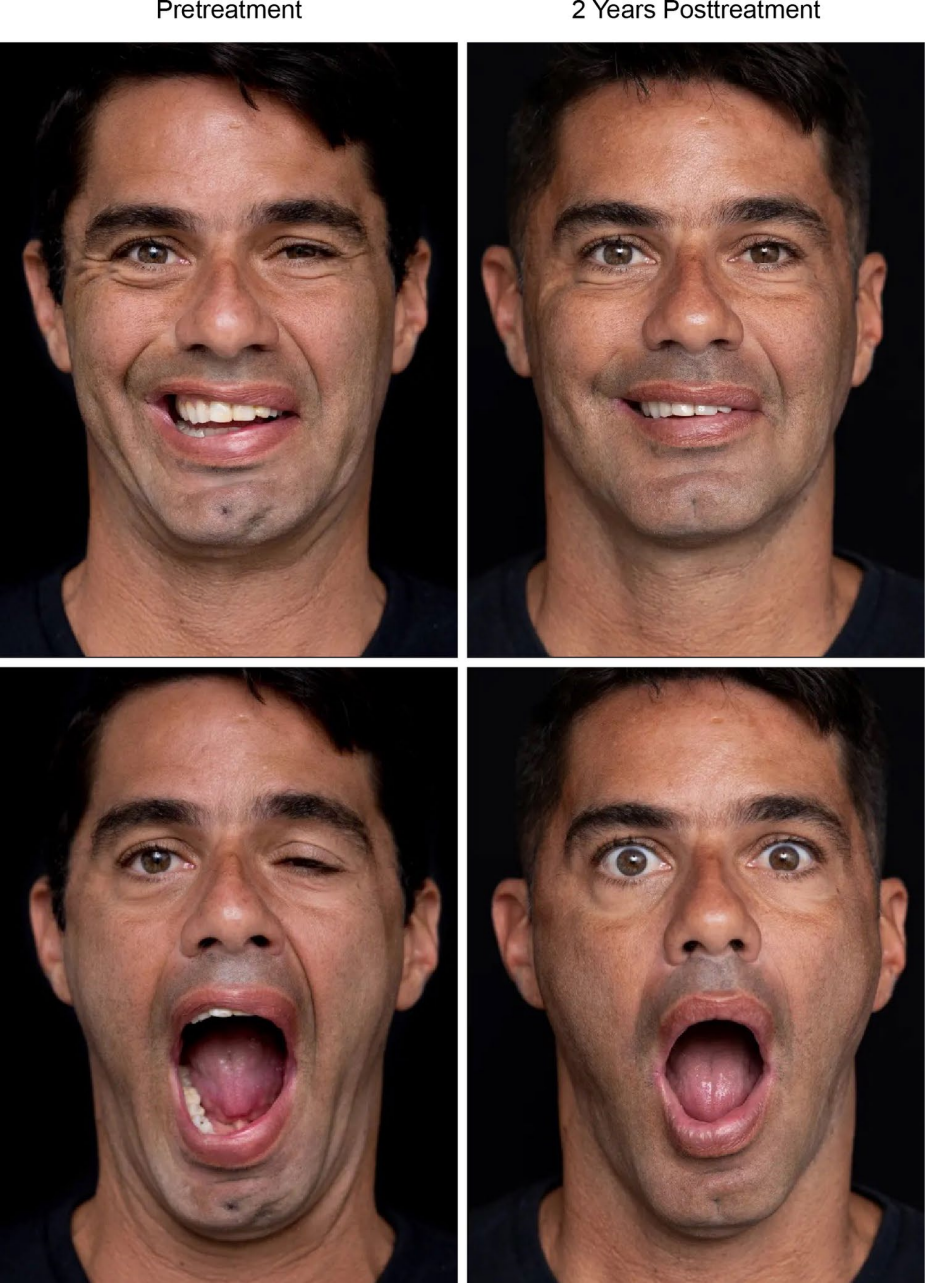
Facial symmetry enhancement in a man with facial paralysis after a herpes zoster infection. Before and after photographs of a 44-year-old White male with Fitzpatrick skin type III, who presented with facial paralysis 3 years after a herpes zoster infection. Clinical examination revealed incomplete movements of the left face, synkinesis, hemifacial spasms, and sequelae of paralysis. HA gels (VYC-15, VYC-17.5, VYC-20, and VYC-25), onabotulinumtoxinA, and a hybrid injectable (HA and calcium hydroxyapatite) were used to remodel facial contour and restore lost volumes. Substantial improvements in facial symmetry were observed posttreatment, with the patient expressing significant satisfaction and increased confidence. No adverse events or complications were reported. Photographs courtesy of Dr. Coimbra. *HA* hyaluronic acid

Substantial improvements in facial symmetry were observed after treatment (Fig. 1). The patient and his family were highly satisfied. He reported resuming eye contact with others, attending social events, and creating content for his social media platforms. Photographs and videos were used to track progress. No adverse events or complications were reported.

#### Correction of Congenital Facial Paralysis

A Hispanic woman 58 years of age with Fitzpatrick skin type III presented with congenital left facial paralysis and a history of hypothyroidism post-thyroidectomy due to cancer. She was disease-free and had no previous aesthetic treatments or other relevant clinical information. Upon clinical examination, the patient exhibited hemifacial asymmetry, eye opening asymmetry, glabellar depression on the right side, a lower right eyebrow, low projection of the middle left cheek, a lower left corner of the mouth, reduced volume in the superior and inferior left lip, deviation of the nasal axis to the right, a deeper right nasolabial fold, and severe dynamic asymmetry when blinking, smiling, speaking, raising eyebrows, and exposing teeth.

The HA gels VYC-17.5L, VYC-20L, and VYC-25L were used to restore the volume lost and facial symmetry. The treatment plan included several interventions. On the left side, 0.3 mL VYC-20L was injected into the malar arch using a needle; 0.5 mL VYC-20L was injected into the deep malar fat pad using a cannula; 0.5 mL VYC-25L was injected in the supraperiosteal plane into the prejowl area and the temporal crest using a needle; 0.5 mL VYC-17.5L was injected into the periorbital area via cannula; 0.2 mL VYC-17.5L was injected into the corner of the mouth using a needle; and 0.3 mL VYC-17.5L was injected into the lips using a needle. On the right side, 1 mL VYC-25L was injected into the temporal area using a supraperiosteal needle; 0.3 mL VYC-20L was injected into the suborbicularis oculi fat and deep anterior malar fat pad; 0.5 mL VYC-20L was injected into the deep nasolabial fold using a supraperiosteal needle; 0.5 mL VYC-20L was injected into the superficial nasolabial fold using a cannula; 0.3 mL VYC-17.5L was injected into the periorbital area via cannula; 0.2 mL VYC-17.5L was injected into the corner of the mouth; and 0.5 mL VYC-17.5L was injected over the upper lip using a superficial cannula. These HA gels were administered in 3 treatment sessions.

Additionally, onabotulinumtoxinA was administered intramuscularly using a 32-G needle for the treatment of hyperkinetic lines. On the left side, 2 U was injected into the depressor anguli oris. On the right side, 10 U was injected in the high frontalis area; 5 U (in total) within the procerus, nasalis muscle, and depressor supercilii; 5 U in the corrugator; and 10 U in the periocular area across 3 points. The total dose was 32 U from 100 U diluted in 1 mL of saline solution. The onabotulinumtoxinA treatments were repeated approximately once every 5 months over a 2-year period.

**Fig. 2:**
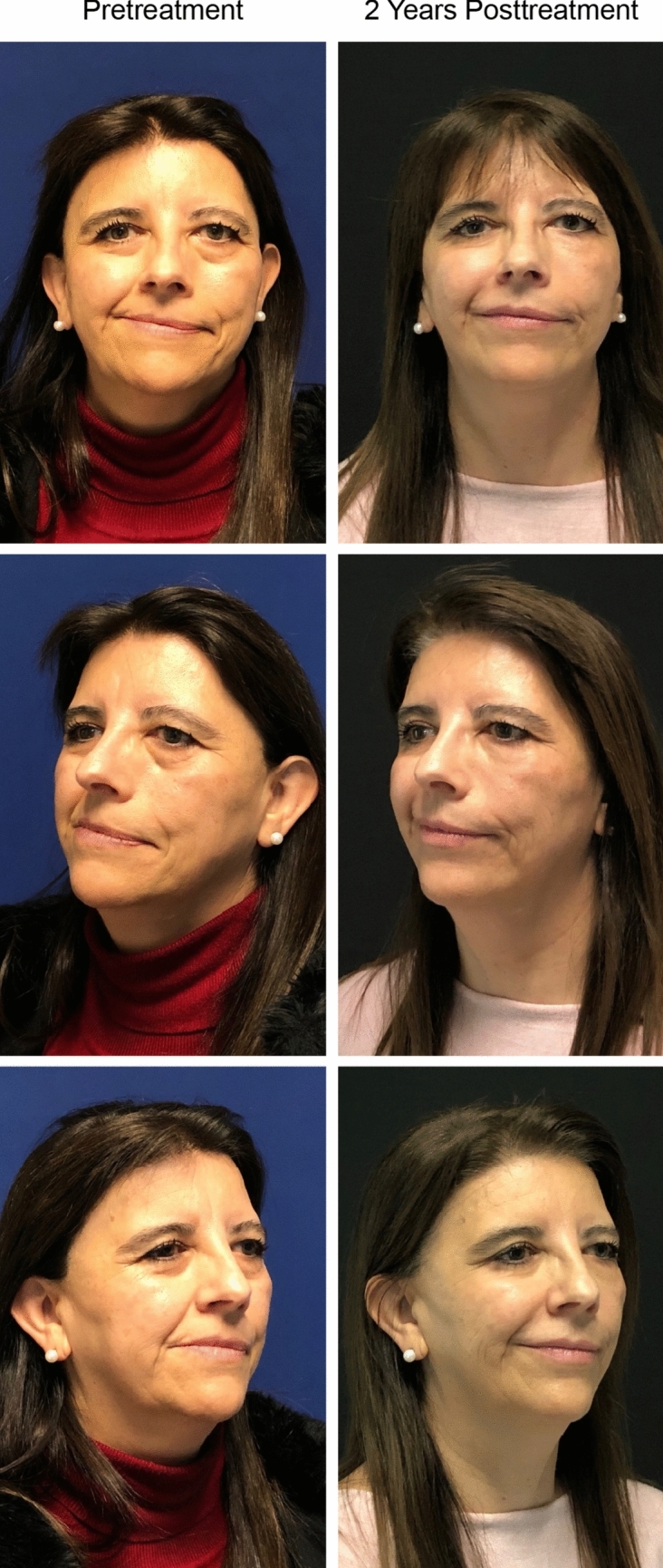
Facial symmetry enhancement in a woman with congenital facial paralysis. Before and after photographs of a 58-year-old Hispanic female with Fitzpatrick skin type III, who presented with congenital left facial paralysis and a history of hypothyroidism post-thyroidectomy due to cancer. Clinical examination revealed hemifacial asymmetry, eye opening asymmetry, glabellar depression on the right side, a lower right eyebrow, low projection of the middle left cheek, a lower left corner of the mouth, reduced volume in the superior and inferior left lip, deviation of the nasal axis to the right, a deeper right nasolabial fold, and severe dynamic asymmetry when blinking, smiling, speaking, raising eyebrows, and exposing teeth. HA gels (VYC-17.5L, VYC-20L, and VYC-25L) and onabotulinumtoxinA were used to remodel the facial contour and restore lost volumes. Improvements in facial symmetry were observed posttreatment, with no adverse events or complications reported. Photographs courtesy of Dr. Villafranca. *HA*, hyaluronic acid

Improvements in facial symmetry were observed after treatment (Fig. 2). Photographs were used to track progress. No adverse events or complications were reported.

#### Correction of Hemifacial Microsomia Type I

A White woman 20 years of age with Fitzpatrick skin type III presented with hemifacial microsomia type I. The patient had no comorbidities or previous treatments. Clinical examination revealed secondary facial asymmetry due to mild hypoplasia of the soft tissues of the left hemiface; mild hypoplasia of the ramus, angle, and body of the left mandible; asymmetric malar hypoplasia predominantly on the left hemiface; and bilateral moderate nasojugal folds.

The HA gels VYC-15L, VYC-20L, and VYC-25L were used to restore facial symmetry. The anatomical areas treated were the tear troughs, cheeks, mandible, chin, and nose. The injection technique involved fanning suborbicular for the tear troughs; linear retrograde supraperiosteal, supracondral, and intercrural L-frame for the nose; and supraperiosteal multiplane columns for the other areas. The needle gauge was 30 G for all areas except the nasojugal folds and nose, which were treated with a 27-G cannula. The volumes injected are listed in Table 2. These HA gels were administered in 2 treatment sessions, first for facial symmetry and 2 months later for the nose. No botulinum toxins were used during treatment.

**Table 2:** Injection areas and HA gel volumes/types used to treat hemifacial microsomia type I

Injection Area	HA Gel Volume (mL)			HA Gel Type
	Left	Midline	Right	
Zygomatic arch	0.4	–	0.2	VYC-20L
Zygomatic eminence	0.6	–	0.4	VYC-20L
Anteromedial cheek	0.3	–	–	VYC-20L
Nasojugal crease	0.6	–	0.4	VYC-15L
Mandible angle	0.6	–	–	VYC-20L
Mandible ramus	0.4	–	–	VYC-20L
Lower lateral chin	–	–	0.5	VYC-20L
Lower anterior chin	–	–	0.2	VYC-20L
Parasagittal pogonion	0.4	–	0.4	VYC-20L
Parasagittal chin	–	–	0.4	VYC-20L
Lateral chin	–	–	0.2	VYC-20L
Nose	–	1.0	–	VYC-25L

*HA* hyaluronic acid

**Fig. 3:**
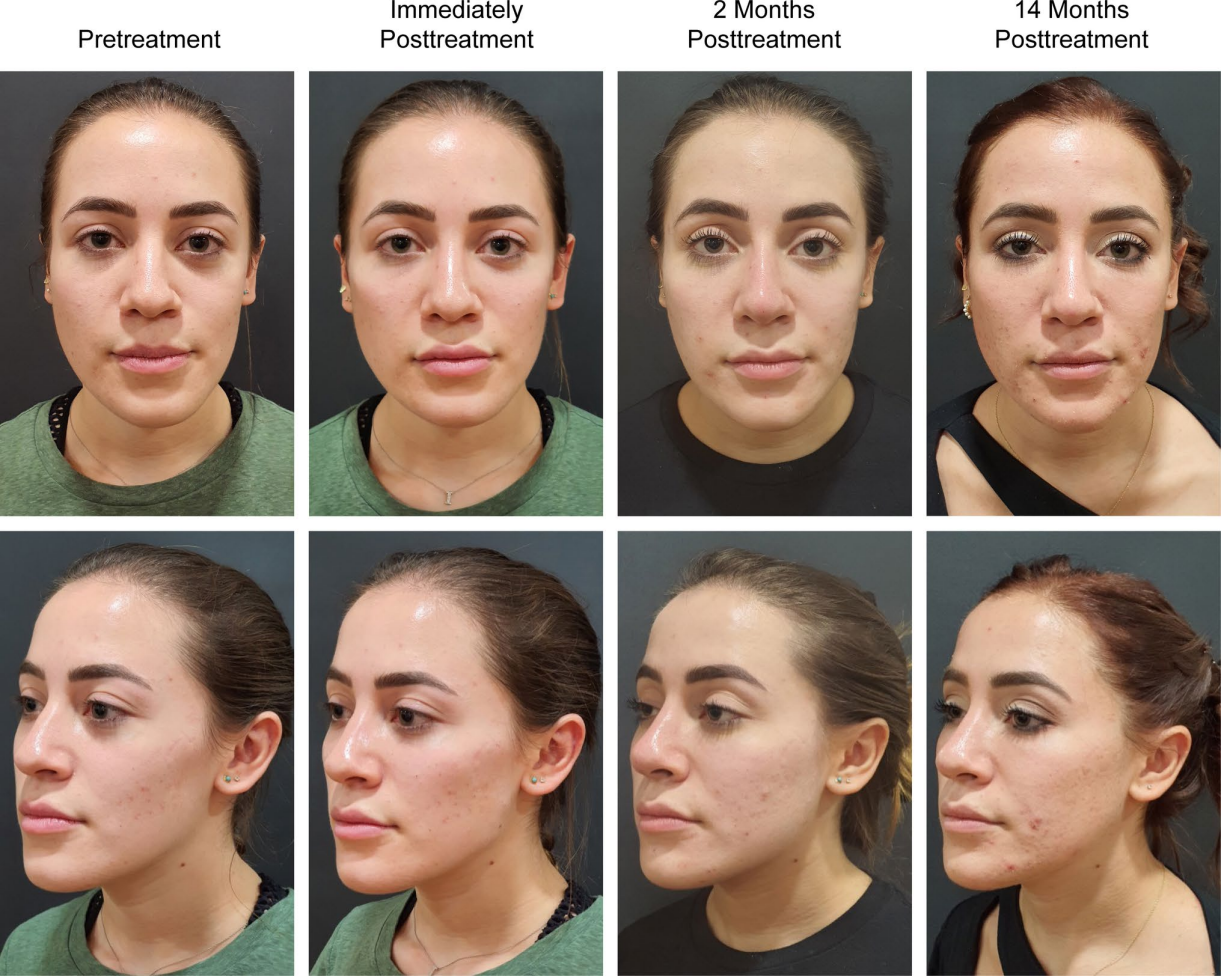
Facial symmetry enhancement in a woman with hemifacial microsomia. Before and after photographs of a 20-year-old White female with Fitzpatrick skin type III, who presented with hemifacial microsomia type I. Clinical examination revealed secondary facial asymmetry due to mild hypoplasia of the soft tissues of the left hemiface; mild hypoplasia of the ramus, angle, and body of the left mandible; asymmetric malar hypoplasia predominantly on the left hemiface; and bilateral moderate nasojugal folds. HA gels (VYC-15L, VYC-20L, and VYC-25L) were used to remodel the facial contour and restore lost volumes. Significant improvements in facial symmetry were observed posttreatment, with no adverse events or complications reported. Photographs courtesy of Dr. Shturman. *HA*, hyaluronic acid

Improvements in facial symmetry were observed after treatment (Fig. 3). Photographs were used to track progress. No adverse events or complications were reported.

### Scar Correction

#### Correction of Scarring Resulting from Cancer Treatments

A Hispanic woman 44 years of age with Fitzpatrick skin type IV presented with a history of synovial sarcoma in the left paranasal sinus diagnosed 2 years earlier. She underwent a left radical maxillectomy followed by chemotherapy and radiotherapy. Reconstructive treatments included an iliac crest flap and muscle graft. Clinical examination revealed changes in facial contour and variations in anatomy due to the surgical and oncological procedures, with marked adhesions of deep planes, irregularities, and loss of facial volumes. She was in remission and disease-free at the time of HA gel treatment.

The HA gels VYC-15L, VYC-17.5L, and VYC-20L were used to remodel the facial contour and recover lost volumes. In the first session, the midfacial structure in the left zygomatic arch was treated with 0.1 mL VYC-20L in 3 deposits using a supraperiosteal needle injection method. The anteromedial malar region received 0.3 mL VYC-17.5L using a fan deposit technique with a 22/40 cannula. Additionally, the left submalar area (buccal area) was treated with 0.6 mL VYC-20L using a fan retro injection deposit with a 22/40 cannula. In the second session, anteromedial remodeling and the nasojugal sulcus in the superficial plane (subdermal) were treated with 0.4 mL VYC-15L using a fan deposit technique with a 22/40 cannula. The left submalar area (buccal area) received another 0.6 mL fan retro injection deposit of VYC-20L using a 22/40 cannula. Additionally, the left piriform area and myomodulation of the orbicularis muscle of the upper lip were addressed with a retro injection deposit of 0.2 mL VYC-15L in the superficial piriform space and 0.2 mL in the subdermal plane of the upper lip for myomodulation. No botulinum toxins were used during the treatment.

**Fig. 4:**
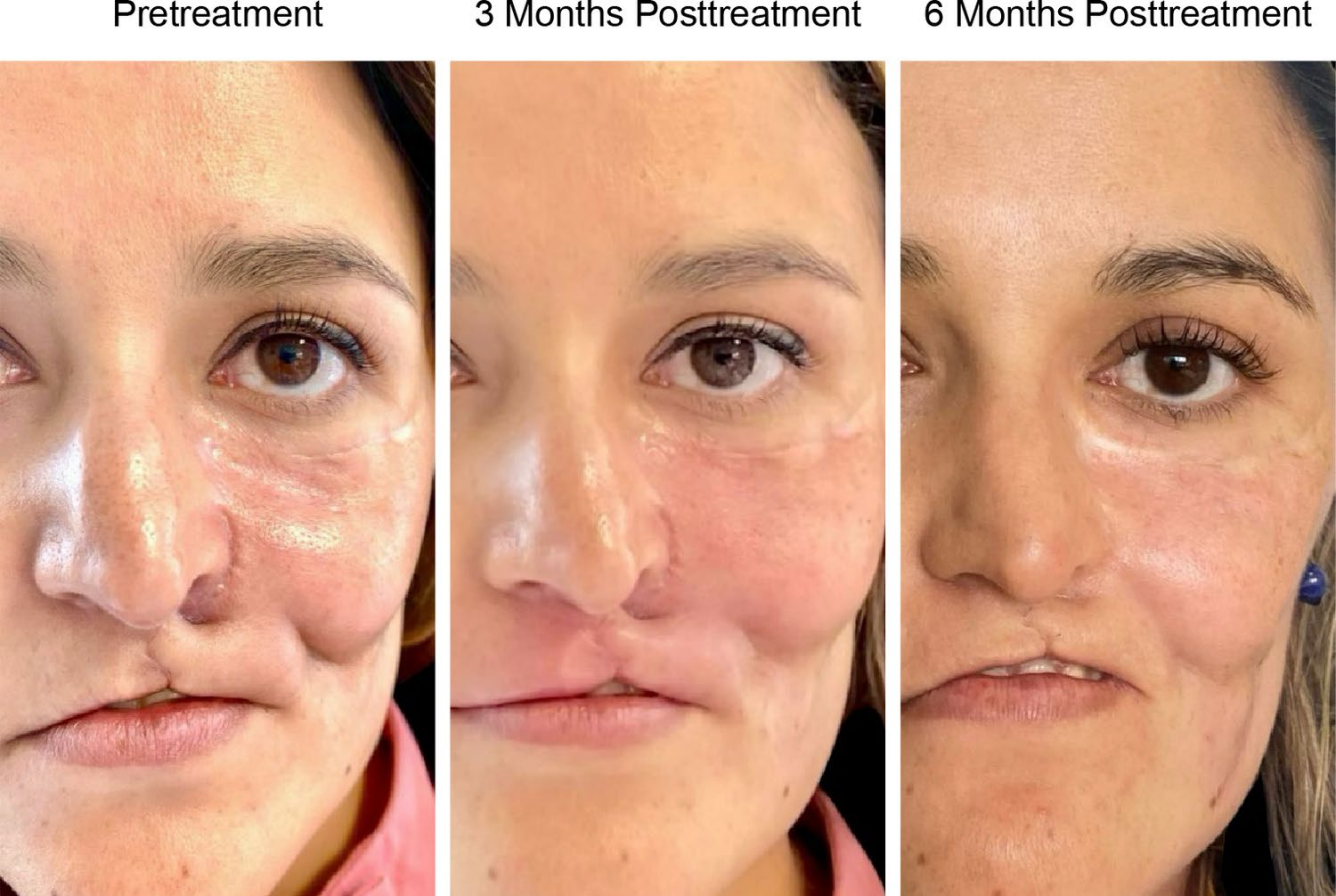
Facial symmetry and contour remodeling after synovial sarcoma treatment. Before and after photographs of a 44-year-old Hispanic female with Fitzpatrick skin type IV, who presented with significant facial contour changes after a left radical maxillectomy, chemotherapy, and radiotherapy for synovial sarcoma. Reconstructive treatments included an iliac crest flap and muscle graft. HA gels (VYC-15L, VYC-17.5L, and VYC-20L) were used to remodel the facial contour and restore lost volumes. Significant improvements in facial symmetry were observed posttreatment, with no adverse events or complications reported. Photographs courtesy of Dr. Garzón. *HA*, hyaluronic acid

Improvements in facial symmetry and contour were observed after treatment (Fig. 4). Photographs were used to track progress. No adverse events or complications were reported after the treatments.

#### Correction of Facial Trauma Caused by an Accident

A White woman 44 years of age with Fitzpatrick skin type III presented with a history of a bicycle accident in 2020 resulting in a fracture and facial sinking. She underwent reconstruction with oral and maxillofacial surgery, which led to partial facial nerve sequelae. Clinical examination revealed temporal deflation, deep tear troughs, accentuated nasolabial folds, a retracted chin, medial malar deflation, orbicularis hypercontraction of the eyes, zygomatic hypercontraction, a gummy and asymmetric smile, and platysma hypercontraction. The patient had no comorbidities and had not received any previous treatments other than the surgery.

The HA gels VYC-15L, VYC-17.5L, VYC-20L, and VYC-25L were used to remodel the facial contour and recover lost volumes. The anatomical areas treated were the temples, lateral orbital, cheeks, nasolabial folds, mandible, and chin. The injection technique involved supraperiosteal and subcutaneous methods with needles and 22-G or 25-G 48-mm cannulas. The HA gel and volumes for each injection area are listed in Table 3. These HA gels were administered in a single treatment session. No botulinum toxins were used during treatment.

**Table 3:** Injection areas and HA gel volumes/types used to treat facial trauma caused by a bicycle accident

Injection Area	HA Gel Volume (mL)			HA Gel Type
	Left	Midline	Right	
Anterior and posterior temples	0.5	–	0.5	VYC-20L
Lateral infraorbital and central lateral orbital	0.4	–	0.2	VYC-15L
Zygomatic arch	0.5	–	0.4	VYC-20L
Anteromedial cheek	0.5	–	0.5	VYC-17.5L
Zygomatic eminence	0.3	–	0.1	VYC-20L
Submalar/buccal area	–	–	0.5	VYC-17.5L
	0.5	–	–	VYC-20L
Upper, central, and lower nasolabial folds	–	–	0.5	VYC-17.5L
	0.7	–	–	VYC-20L
Prejowl area	0.5	–	0.5	VYC25L
Mandible body	0.5	–	0.5	VYC25L
Mental crease	0.3	–	0.3	VYC-20L
Chin apex	0.2	–	0.2	VYC-20L
Anterior chin	0.2	–	0.2	VYC-25L

*HA* hyaluronic acid

**Fig. 5:**
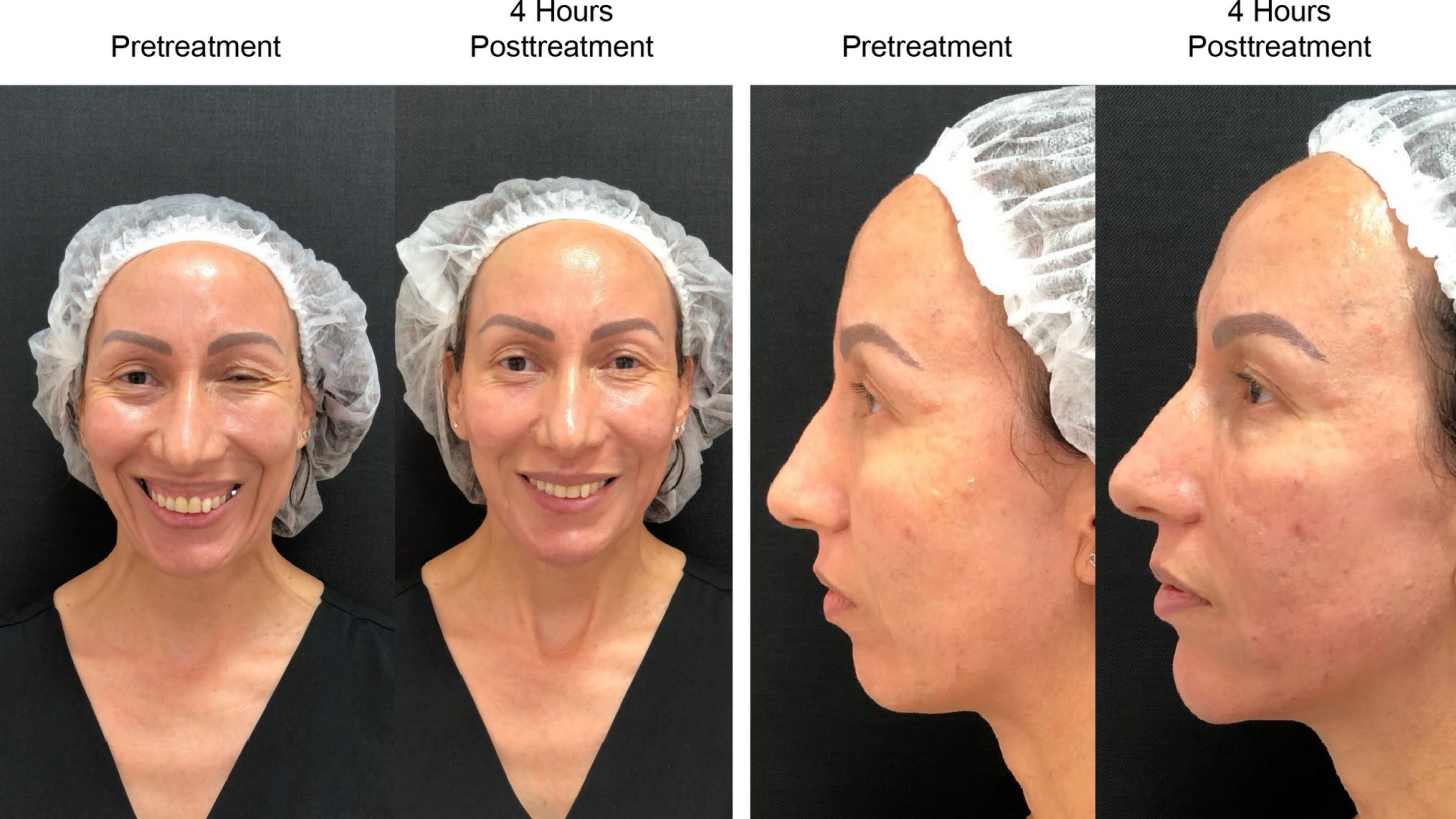
Facial symmetry improvement after facial trauma via bicycle accident. Before and after photographs of a 44-year-old White female with Fitzpatrick skin type III, who presented with facial asymmetry and other sequelae after a bicycle accident in 2020. The patient underwent reconstruction with oral and maxillofacial surgery, leading to partial facial nerve sequelae. Clinical examination revealed temporal deflation, deep tear troughs, accentuated nasolabial folds, a retracted chin, medial malar deflation, orbicularis hypercontraction of the eyes, zygomatic hypercontraction, a gummy and asymmetric smile, and platysma hypercontraction. HA gels (VYC-15L, VYC-17.5L, VYC-20L, and VYC-25L) were used to remodel the facial contour and restore lost volumes. Improvements in facial symmetry and expression were observed posttreatment, with no adverse events or complications reported. Photographs courtesy of Dr. Zimbres. *HA* hyaluronic acid

Improvements in facial symmetry were observed after treatment (Fig. 5). Improvements were noted in eye aperture, chin projection, the gummy smile, and platysma contraction. Overall, the patient reported marked improvements with HA gel when compared with many years of physiotherapy. Photographs were used to track progress. Mild bruising was observed, which resolved spontaneously after 7 days. No other adverse events or complications were reported.

### Treatment Considerations

Minimally invasive treatments with onabotulinumtoxinA and HA gels are known to be effective and generally tolerable, with mostly transient, mild to moderate adverse events (Tables 4 and 5) [2, 3, 17, 45–53]. General precautions are important for optimizing outcomes with facial injectable procedures with onabotulinumtoxinA and HA gels, including a thorough understanding of facial anatomy, performing careful patient selection, educating patients, using the appropriate type and amount of gel product, employing good injection and aseptic techniques, and providing sufficient postprocedural care [6, 54–58]. Importantly, special considerations should be given to how facial vasculature may vary in corrective archetype patients based on preexisting conditions [59].

**Table 4:** Managing adverse events associated with botulinum toxin treatment

Clinical Signs	Possible Cause	Recommended Therapy
Erythema, ecchymosis, bruising, hematoma	Injuring a blood vessel	Tamponade, pressure, ice packs
Tenderness and pain at the site of injection, injection trauma	The needle puncturing the skin	Topical anesthetic creams
Dry skin	Decreased sweat gland activity	Emollients
Local infection, abscess	Contamination of the injection site	Course of antibiotics
Paresthesia or dysesthesia	Nerve injures	No specific treatment
“Mephisto sign,” exaggeration of wrinkles, brow ptosis, periorbital edema	Aesthetic in periorbital area	Paralysis of antagonist muscles
Blepharoptosis, ptosis ectropion, dry eyes, corneal irritation, corneal exposure, blurred vision, accommodation difficulties, retinal detachment, diplopia, strabismus	Functional in periorbital area	α-Adrenergic drops, topical lubricating drops, ophthalmologic consultation
Asymmetric smile, lip ptosis, changes in facial expression	Aesthetic in perioral area	No specific treatment
Trismus	Functional in perioral area	Mechanotherapy
Xerostomia		Sialagogues
Redness, edema, urticaria	Localized allergic reaction	Antihistamine drugs, local steroids
Generalized urticaria, diffuse edema, anaphylactic shock	Generalized allergic reaction	Steroids, adrenalin injection, monitoring of patients
Headache	Other mechanism	Analgesics
Severe dysphagia, generalized muscle weakness	Diffuse spread of toxin	Neurotrophic drugs, systemic support, and symptomatic treatment botulinum antitoxin serum

Adapted with permission from Witmanowski et al [52] under the Creative Commons Attribution-NonCommercial-ShareAlike 4.0 International (CC BY-NC-SA 4.0) License.

**Table 5:** Managing adverse events associated with HA filler treatment

Clinical Signs	Possible Cause	Recommended Therapy
Whitening/blanching of the skin and pain, followed by livedo	Vascular occlusion (intravascular/extravascular, complete/partial)	Hyaluronidase, warmth, anticoagulation; if necessary, further rheological and surgical procedures
Swelling	Injection trauma, hematoma	Depending on trauma pressure, cooling
Angioedema-like swelling (mainly in the lip area)	Allergic or anaphylactic reaction	Observation: if no muco-cutaneous involvement, usually will resolve in approximately 1 to 2 h. If persisting or muco-cutaneous involvement: intravenous antihistamines/steroids, see “Anaphylaxis”
Signs of systemic anaphylaxis	Anaphylaxis (e.g., lidocaine, BDDE)	Anaphylaxis treatment (including epinephrine injection and emergency procedures)
Neurological symptoms, e.g., impairment of vision	Retrograde embolization of central arteries with ischemia, e.g., ophthalmic artery	Emergency treatment at site or at neurological/ophthalmic stroke unit, e.g., systemic anti-ischemia treatment, retrobulbar hyaluronidase injection
Swelling without discoloration	Injection trauma, hygroscopic effect	Observation: may be homoeopathic drugs or bromelain-containing drugs
Swelling with dark or livid discoloration	Hematoma	Heparin-/vitamin K–containing creams
Livedo (reddish- or blue-mottled discoloration)	Vascular obliteration (intravascular/extravascular, complete/partial)	Hyaluronidase, warmth, anticoagulation; if necessary, further rheological and surgical procedures
Newly developed redness and swelling (with signs of inflammation)	Infection	Antibiotics
Newly developed fluctuating single process (with signs of inflammation)	Abscess	Incision, drainage, antibiotics
Persisting swelling without signs of inflammation or skin discoloration	Injection material–dependent: swelling due to hygroscopic effect, wrong injection site, or overdose	Hyaluronidase
New swelling without signs of inflammation or skin discoloration	Migration of injection material or reaction to injection material	Hyaluronidase, if no response to steroids
Single knot/swelling/induration (possible slight redness)	Chronic inflammation, possible biofilm	Antibiotics; if no response, hyaluronidase in addition, steroids if no response to the previous
Multiple knots/swelling/indurations (possible slight redness)	Possible biofilm (look for signs of previous infection, dental treatment, etc.). Also consider chronic immunological reactions (granuloma)/chronic systemic diseases	Antibiotics; if no response, hyaluronidase in addition (steroids or other immunosuppressives only if no response to the previous or if histopathological proof of granuloma)

Adapted with permission from Philipp-Dormston et al [53]. Copyright 2017 John Wiley and Sons. All rights reserved. License number 6101350190644.

Although rare, serious adverse events can occur with HA gels (e.g., blindness and vascular occlusion), and patients should be aware of these and other potential complications [2]. Complications are typically associated with HA gel product placement, volume, sterility, injection technique, and/or the patient’s clinical history, current medications, and prior interventions, but complications are mostly avoidable with proper planning and technique [6, 54]. Proper understanding of the signs and symptoms associated with injectable complications is also important for minimizing the progression of adverse events, as well as having materials on hand to treat any complications that arise [54, 59]. For example, hyaluronidase should be readily available if needed to correct HA gel misplacement and vascular occlusion [53, 57, 60].

Cosmetic procedures may generally be discouraged, even if not contraindicated, for some treatments/conditions (e.g., active chemotherapy, rheumatoid arthritis, systemic lupus erythematosus, and others); however, healthcare professionals should consider the psychological and functional impacts that these conditions may have on each patient, which can ultimately cause loss of identity [58]. In such cases, proceeding with cosmetic procedures should be considered to support the patient’s overall well-being. Additionally, psychological evaluations may be warranted for certain patients to ensure the potential benefits of procedures outweigh any risks (such as those described in Tables 4 and 5). It is crucial to conduct a collaborative, interdisciplinary, comprehensive risk–benefit assessment and clearly communicate all potential risks before performing any cosmetic procedures [58]. In general, caution should be taken with regard to all off-label uses of products with limited data, and thorough and comprehensive follow-ups should be performed to address the patients’ needs safely and effectively.

## Discussion

### Overview of Clinical Outcomes

This manuscript presents a descriptive review of the literature accompanied by illustrative clinical examples that show minimally invasive injectable treatments were associated with consistent improvements in facial symmetry, contour, and dynamic balance in patients within the corrective patient archetype. Across diverse etiologies, including congenital conditions, post-infectious paralysis, trauma, and oncologic reconstruction, individualized treatment approaches using onabotulinumtoxinA, HA gels, and hybrid injectables effectively addressed both static and functional asymmetries. Clinical outcomes were accompanied by high patient satisfaction and a favorable safety profile, with adverse events limited to mild and transient effects, supporting the role of injectable therapies as a viable option for corrective facial treatment in complex clinical scenarios.

### Clinical Perspectives

Achieving facial balance with onabotulinumtoxinA and HA gels requires a nuanced understanding of both anatomy and muscle dynamics, particularly in cases of asymmetry or post-paralytic imbalance. Treatment decisions, such as which muscles to inject, which to avoid, and how to tailor concentration, are guided not only by aesthetic goals but also by functional considerations. Muscles involved in expressive movement, such as the depressor anguli oris, zygomaticus, orbicularis oculi, and elevators of the oral commissure, demand precise technique, and clinically appropriate dosing. Over-treatment in these areas can lead to a flat, sagging, or inexpressive appearance, while under-treatment may fail to correct asymmetry or hyperactivity. In addition, the choice of injection plane, whether supraperiosteal or superficial, affects both contour and muscle modulation. Deep placement can provide structural support and leverage beneath musculature, while superficial placement can add weight to temper excessive contraction. Dose asymmetry may be necessary to balance hyperdynamic muscles on one side with weaker or less active muscles on the other. Ultimately, successful outcomes depend on a comprehensive technical and aesthetic assessment of both symmetric and asymmetric regions, with the goal of restoring harmony, enhancing expression, and avoiding unintended functional compromise.

### Strengths and Limitations

The strengths of these clinical examples are the relevant insights into the use of minimally invasive injectable treatments for patients within the corrective patient archetype, a population frequently encountered in practice yet underrepresented in controlled studies. The clinical examples illustrate real-world corrective scenarios across congenital, post-infectious, traumatic, and oncologic etiologies and highlight the importance of individualized, multimodal treatment strategies using injectables that offer flexibility and adaptability in patients with altered anatomy. In addition to improvements in facial symmetry and contour, outcomes included functional balance and patient-reported psychosocial benefits. Limitations include the descriptive nature of the work, the small number of clinical examples, lack of standardized quantitative outcome measures, heterogeneous indications and treatment approaches, and variable follow-up duration, which limit generalizability and preclude statistical comparison.

## Conclusions

OnabotulinumtoxinA, HA gels, and hybrid injectables are effective and well-tolerated treatments for corrective aesthetic procedures. The combination of onabotulinumtoxinA and HA gels may enhance the results of patients seeking corrective treatments. These treatments can be positively impactful and transformative by addressing both aesthetic and functional concerns. These descriptive cases emphasize the importance of multimodal tailored approaches, a thorough understanding of facial anatomy and underlying conditions, patient concerns determined via consultation, and careful patient assessment and selection to optimize outcomes. It also highlights the need for further clinical research in the disease states discussed here to refine these treatments and expand their applications. Overall, onabotulinumtoxinA, HA gels, and hybrid injectables play a crucial role in improving psychological and social outcomes for patients seeking corrective aesthetic treatments.

## Data Availability

AbbVie is committed to responsible data sharing regarding the clinical trials we sponsor. This includes access to anonymized, individual, and trial-level data (analysis datasets), as well as other information (e.g., protocols, clinical study reports, synopses, or statistical analysis plans), as long as the trials are not part of an ongoing or planned regulatory submission. These clinical trial data can be requested by any qualified researchers who engage in rigorous, independent, scientific research, and will be provided following review and approval of a research proposal and Statistical Analysis Plan (SAP) and execution of a Data Use Agreement (DUA). Data requests can be submitted at any time after approval in the USA and Europe and after acceptance of this manuscript for publication. The data will be accessible for 12 months, with possible extensions considered. For more information on the process or to submit a request, visit the following link: https://vivli.org/ourmember/abbvie/ then select “Home”.
